# Cervical microendoscopic laminoplasty-induced clinical resolution of disc herniation in patients with single- to three-level myelopathy

**DOI:** 10.1038/s41598-022-23747-z

**Published:** 2022-11-07

**Authors:** Chunlin Zhang, Su Fu, Xu Yan, Dongzhe Li, Yongming Ning, Chao Dong, Yongkui Wang, Yang Feng

**Affiliations:** Department of Orthopaedics, The First Affiliated Hospital of Zhengzhou University, Zhengzhou, 450000 China

**Keywords:** Clinical trials, Neurological disorders, Neuropathic pain, Musculoskeletal system

## Abstract

This study aimed to explore the effects on resorption of cervical disc herniation (CDH) and clinical outcomes of surgery. Cervical microendoscopic laminoplasty (CMEL), which is commonly preferable to anterior corpectomy and fusion, was applied to patients with 1- to 3-level degenerative cervical myelopathy (DCM). DCM patients with 1–3 levels DCM underwent either conservation treatment or CMEL. In conservation-treated patients (53 cases), CDH volume remained unchanged with no improvement in JOA and VAS scores. Conversely, 63 patients with 1–3 levels DCM were prospectively enrolled and exhibited a profound decrease in CDH volume: 89.1% of CDHs (123/138) regressed over 10%, 64.5% of CDHs (89/138) regressed over 25%, while 27.5% and 6.5% of CDHs (38/138 and 9/138) largely regressed over 50% and 75%, respectively. Meanwhile, the JOA and VAS scores were improved in different ways. Intriguingly, CDH volume changes correlated significantly with elevations in JOA scores, indicating an association of clinical CDH resolution with neurological recovery. We showed that CMEL induced clinically related diminishment of CDH and alleviation of clinical symptoms in patients with 1- to 3-level myelopathy and that it could help avoid anterior dissection of the disc to some extent.

## Introduction

Degenerative cervical myelopathy (DCM) represents the leading cause of spinal cord dysfunction in elderly individuals^1^ and is characterized by a degenerative process with cervical disc herniation (CDH) leading to stenosis of the spinal canal and resultant cord compression. As the natural history of CDH offers only a limited capacity for spontaneous resorption^2^, surgical treatment is regarded as the most effective option. The optimal treatment has not been established^3^ but depends on the specific patient. Among major surgical operations, anterior cervical corpectomy and fusion allow direct removal of CDH, whereas posterior laminoplasty facilitates enlargement of the spinal canal with preservation of CDH. Ideally, the indications for laminoplasty refer to multilevel stenosis (3 or more segments)^4^, while indications for corpectomy and fusion include myelopathy of one to three levels^5^.

Recently, we proposed the clinical application of modified laminoplasty, namely, cervical microendoscopic laminoplasty (CMEL)^6^, in patients with 3 or more levels of myelopathy. To reduce the complications of popular laminoplasty operations such as neck pain and restricted range of motion^7^, we performed an invasive technique that symmetrically expanded the laminar arch, preserving the posterior spinous process-ligament complex and the deep extensor muscles. This technique showed promising improvement of neurologic deficits and pain and the final bone healing of decompression groove fragments, restoring the original spinal canal anatomy^6^.

Interestingly and clearly, a common occurrence of spontaneous resorption of CDH was seen by our team^8^ in patients with multiple levels of myelopathy after CMEL treatment. Therefore, CMEL was hypothesized to effectively decompress nerves by both enlarging the spinal canal and, importantly, further inducing CDH resolution. Our retrospective observation of multilevel myelopathy inspired this prospective study exploring the effect of CMEL in patients with 1- to 3-level DCM. Laminoplasty-induced decompression of the spinal cord is traditionally regarded as causing a shift away from lordotic curvature, favouring kyphosis in which laminoplasty is contraindicated^9^. However, recently updated knowledge has suggested that shifting of the spinal cord posteriorly is largely correlated with the local shifting of the dural sac^10^ and not so much the cervical curvature index^11^, justifying our study design.

As a consequence, whether CMEL still effectively induces the resolution of CDH and results in better clinical outcomes in patients with 1- to 3-level DCM is largely unknown. This present prospective study enrolled 116 DCM patients with 1- to 3-level myelopathy and/or stenosis, which is commonly the preferred indicator for anterior surgery. The patients prospectively underwent CMEL and then had follow-up involving MRI detection and evaluation of functional recovery.

## Methods

### Patient population

DCM patients of levels 1–3 underwent conservation or CMEL. In this study, conservation-treated patients were retrospectively followed-up with MRI detection and outcome assessment. Patients with 1–3 levels DCM were prospectively enrolled in a study and randomly divided into groups, regardless of the number of myelopathy levels. The trial described the clinical outcomes of CMEL in patients with 1–3 levels DCM, thus addressing out our hypothesis but without a control group. We, therefore, retrospectively surveyed conservation-treated patients with symptoms similar to those of CMEL patients as controls. The patients who received conservative treatment were first given an opportunity to receive surgical treatment in the same institution. They understood but refused due to personal reasons, mostly because of fear of surgery and medical costs. Conservative treatment included use of the nonsteroidal anti-inflammatory drug etoricoxib to control pain, cervical collar fixation, and physical therapy to strengthen and stretch the neck.

Briefly, prospective consecutive DCM patients from a single institution with spinal symptoms requiring surgical treatment of CMEL were enrolled from May 2019 to May 2021. This prospective trial was approved by the ethics committee of the First Affiliated Hospital of Zhengzhou University (Research-2019-LW-049) on 21/03/2019 and has been registered at chictr.org.cn with the number ChiCTR1900023100 (11/05/2019). Patients chose CMEL as the method of operation after fully understanding the procedure and giving their signed consent. The final cohort of patients (a total of 116 patients) met the inclusion criteria and were included in this study. Principal inclusion and exclusion criteria are presented in Table 1. The sample size was initially set by the sample size formula, while over half of the number was lost to follow-up postoperatively. The data were collected in our institution. The independent data monitoring committee supervised and managed the data throughout the clinical research process. The patient randomization process was performed according to the method of the random number table in the third part. We also used a blinded method for evaluators who did not participate in managing the patients.

**Table 1:** Inclusion and exclusion criteria for study subjects.

**Inclusion criteria**
1. Age ≥ 20 years
2. Single- to three-level myelopathy at C3–C7
3. All patients received conservative treatment or underwent CMEL performed by a fixed and experienced spinal team
4. Radiological evidence of one to three CDHs by MRI
**Exclusion criteria**
1. Continuous ossification of the posterior longitudinal ligament confirmed by CT
2. No completed initial and follow-up MRI image data
3. Inability to provide informed consent for study participation

### CMEL procedure

As established previously^6^, bilateral decompression achieved by symmetrically pulling the spinal dorsal elements was performed in an endoscope-assisted manner. All steps of the operation were performed by the same surgical team in the same institution. Briefly, a midline skin incision was made to fully explore. After drilling and threading through the spinous process, two holes were drilled at the basilar part of the spinous process directed at the contralateral lamina for further docking of plates. Endoscopically, the connecting part of the lamina and the lateral mass were drilled for full separation. Then, the posterior structure was pulled dorsally 1–3 mm in the sagittal direction through the silk suture located on the spinous process. Two titanium miniplates were finally fixed on both arcs of the lamina with two screws for each plate, thus stabilizing the bone structure. Basically, the levels of laminoplasty were chosen to extend over the range of CDH, e.g., C4-6 for patients with C4/5 and C5/6 herniation. For patients possessing spinal canal stenosis as well, the levels of laminoplasty were expanded for adequate decompression. These situations, such as ligamentum flavum hypertrophy, indicated a combined compressor from the posterior structure. Early-stage rehabilitation was conducted. After surgery, the patient was fitted and equipped with a soft cervical collar during hospitalization until 3 weeks after surgery. Then, they were asked to perform range-of-motion exercises as possible per pain.

### Clinical outcome measurement

This study typically used the Japanese Orthopaedic Association (JOA) scores to grade the severity of cervical myelopathy (full score = 17 points) based on the patients’ preoperative and follow-up function. Data collection was performed by independent reviewers. Each patient’s status was also measured by the visual analogue scale (VAS) score (0 meaning no pain and 10 representing worst imaginable pain) of axial neck pain (nuchal, neck, or shoulder). Throughout the study, the patients were examined by two doctors separately to calculate the mean score, which was then regarded as the final result.

### CDH volume measurement

The CDH volume was calculated based on MRI T2WI images and equalled the sum of each area in the sagittal image multiplied by the scan thickness. The measurement method was consistent with previous studies^12^. The herniated disc area of each layer was measured. In addition, the layer spacing and layer thickness were recorded. Subsequently, the herniated disc volume was calculated as follows: herniated disc volume = (layer spacing + layer thickness) × ∑ the herniated disc area of each layer. This was completed by two independent professional physicians; thus, the manual measurement error was reduced by averaging each physician’s values.

The resolution ratio of CDH volume was calculated using the following formula: resolution ratio (%) = [(preoperative volume − postoperative volume)/preoperative volume] × 100. The ratio of CDH volume change > 75%, 75% to 50%, 50% to 25%, and 25% to 10% was considered complete, massive, partial, and little resolution, respectively; a ratio between −10 and 10% was regarded as unchanged considering the manual error; and a ratio < −10% was considered reprotrusion of CDH.

### Statistical analysis

Statistical analysis was performed using SPSS 21.0 (IBM, USA). The data were expressed as mean ± standard deviation (SD). Clinical outcome or volumetric data from preoperative and follow-up calculations were compared using the paired t test if all the data passed the Shapiro‒Wilk normality test. Alternatively, a paired Wilcoxon nonparametric test or Mann‒Whitney test was undertaken. The relationships between factors were analysed by Spearman correlation analysis. The differences in general information and clinical outcomes were compared by the chi-square test. A value of p < 0.05 was considered statistically significant.

### Statement on consent

All the patient have known this trial and signed the informed consent to receive the treatment of CMEL.

### Ethic statement

This prospective clincial trial protocol was approved by the ethic committee of the first affiliated hospital of zhengzhou university (Research-2019-LW-049). All methods including operation and outcome assessing were carried out in accordance with relevant guidelines and regulations.

## Results

After screening with the inclusion and exclusion criteria at the study institution, a total of 116 consecutive patients suffering from DCM accepted conservative treatment (n = 53) or CMEL operation (n = 63), which were referred to as conservation and CMEL, respectively. As listed in Table 2, patients from the conservation group or CMEL group showed no difference in age (53.3 ± 11.7 vs. 54.9 ± 12.6), sex (45.3% vs. 47.6% in males/all) or duration of DCM symptoms (13.9 ± 11.6 vs. 18.9 ± 27.4). As part of a planned follow-up of approximately one year, the patients returned to our institution and accepted MRI examination for 14.6 ± 12.4 months after conservation or 11.4 ± 14.3 months after CMEL (no significant difference). Of them, 5 and 12 patients had 1-level myelopathy, 20 and 27 patients had 2-level myelopathy, and 28 and 24 patients possessed 3 segments of myelopathy (Table 2).

**Table 2:** Demographic data of patients.

Variable	Conservation group (n = 53)	CMEL group (n = 63)	p value
Age (years)	53.3 ± 11.7	54.9 ± 12.6	0.56
**Gender**			
Male	24 (45.3%)	30 (47.6%)	0.80
Female	29 (54.7%)	33 (52.4%)	
Duration of illness (months)	13.9 ± 11.6	18.9 ± 27.4	0.22
Follow-up time (months)	14.6 ± 12.4	11.4 ± 14.3	0.64
**Level(s) of myelopathy**			
1-level	5	12	0.18
2-level	20	27	
3-level	28	24	

**Figure 1:**
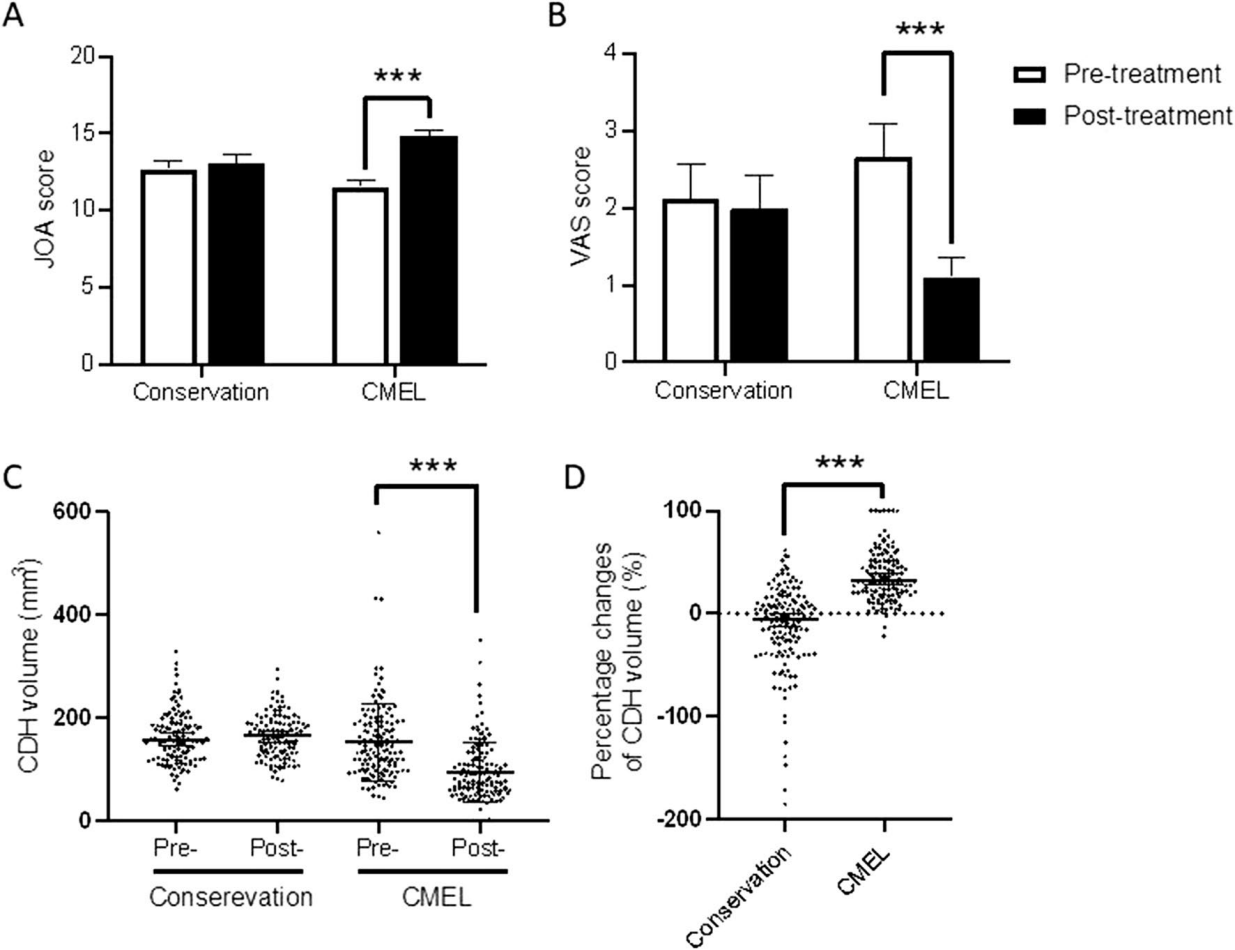
JOA score, VAS score and CDH volume changes in patients treated with either conservation or CMEL. Patients with 1- to 3- levels DCM were treated with either conservation or CMEL. The pre-treatment and post-treatment JOA score (**A**) and VAS score (**B**) were shown. Meanwhile, the CDH volume data (**C**) and change ratios in CDH volume (**D**) were also presented. Paired t test (**A,B**) and paired Wilcoxon test (**C,D**).

The clinical outcomes of patients were assessed using the JOA scoring system (Fig. 1A) and VAS (Fig. 1B). Thirty-two (60.4%) of 53 conservation patients were evaluated, while of 63 patients who received CMEL, 34 were examined (54.0%). Initially, DCM patients suffered from 1- to 3-level myelopathy with JOA scores of 12.7± 2.9 (conservation) and 11.5 ± 2.5 (CMEL), with no difference between groups (Fig. 1A). Conservative treatment did not improve the JOA scores, but CMEL patients recovered with JOA scores of 14.9 ± 1.8 after CMEL operation (p < 0.001, Fig. 1A). Similarly, the VAS scores regarding pain were alleviated from 2.7 ± 2.5 to 1.1 ± 1.4 in CMEL patients (p < 0.001, Fig. 1B) but not in conservation patients (from 2.1 ± 2.5 to 2.0 ± 2.4, p = 0.001, Fig. 1B).

In terms of the alterations in CDH volumes (Fig. 1C), conservation patients did not show a reduction (p = 0.19), but CMEL patients did, which was consistent with our previous study. In detail, the median initial CDH volume was 131.9 mm^3^ (range 39.1–559.7), which significantly decreased to 80.6 mm^3^ (range 0–350.6) in the CMEL group postoperatively (p < 0.001). Further calculation of the percentage changes in CDH volume (Fig. 1D) showed that the median was 3.1% in the conservation (ranging from -61.4% to 147%) group, different from the CMEL median of 31.9% (ranging from -21% to 100%). According to the proposed grading of CDH volume changes (Table 3), 87.4% of CDHs showed resolution (volume regression ratio > 10%); 22.6% of CDHs were resorpted in a small part (10%-25%), while 35.2% of CDHs were resorpted to a median degree (25%-50%); and 29.6% of CDHs were very obviously diminished over half of their initial volume. These data indicated that the surgical treatment of CMEL both improved the recovery of neurological symptoms and reduced CDH size.

**Table 3:** Volumetric changes of CDHs.

CDH resolution	Re-protrude	Unchanged	Partial	Median	Massive	Complete
Conservation (percentages)	60 (47.6%)	18 (14.3%)	28 (22.2%)	16 (12.7%)	4 (3.2%)	0 (0%)
CMEL (percentages)	2 (1.4%)	13 (9.4%)	34 (24.6%)	51 (37%)	29 (21%)	9 (6.2%)

**Figure 2:**
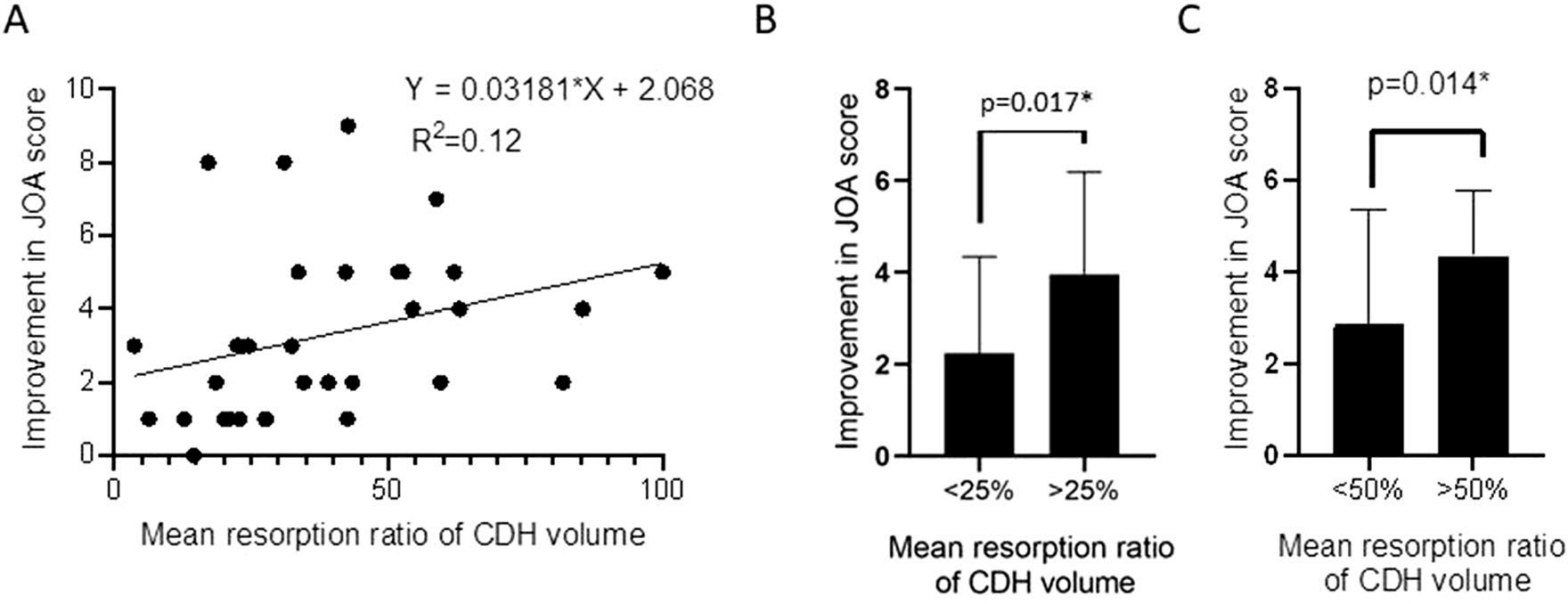
Correlations of change ratio in CDH volume and changes in JOA scores. (**A**) The scatter plots of percentage changes in CDH volume and changes in JOA score demonstrating the significant correlation. The difference in JOA scores of patients between the change ratio of CDH volume < 25% and > 25% (**B**), < 50% and > 50% (**C**).

We next sought to explore the relationships between CDH volume changes and clinical outcomes to clarify the possible clinical meaning of CDH resolution. As shown in Fig. 2A, a positive relationship was found, indicating association of mean change ratio of CDH volume with changes in JOA score. A linear regression (R^2^ = 0.12) was subsequently exported. As shown in Fig. 2B,C, patients with (1) a mean ratio of CDH volume < 25% or > 25%, and (2) a mean ratio of CDH volume < 50% or > 50% both showed a better JOA score/neurological recovery with a larger resorption ratio of CDH.

Subgroup analysis of CMEL patients was also performed to determine whether patients with 1-, 2- or 3-level myelopathy recovered differently after CMEL. There was no difference in demographic information, such as age, sex, and follow-up time (Table 4). All patients showed a similar trend of CDH diminishment (all p < 0.001) and no difference in the percentage changes of CDH volume and the grading distribution of CDH volume reduction (Table 4). Because of the relatively small number of patients with single-level myelopathy, only DCM patients with 2- and 3-level myelopathy showed a significant improvement in JOA and VAS scores (Table 5).

**Table 4:** Demographic data and CDH volume data of CMEL-treated patients.

Variable	Patients with myelopathy			p value
	1-level (n = 12)	2-level (n = 27)	3-level (n = 24)	
Age (years)	45.6 ± 18.9	56.3 ± 11.4	55.4 ± 11.6	0.38
**Gender**				
Male	6	12	12	0.91
Female	6	15	12	
Duration of illness (months)	7.3 ± 9.4	24.5 ± 34.4	15.3 ± 21.4	0.53
Follow-up time (months)	5.8 ± 3.5	10 ± 15.1	10.3 ± 14.6	0.58
Pre-operative CDH volume (mm^3^)	186.2 (92.6–559.7)	135.6 (43–430.4)	120.8 (39.1–297.2)	-
Post-operative CDH volume (mm^3^)	109 (62.1–308)	97.3 (0–350.6)	73.7 (0–170.4)	-
Percentage changes in CDH volume	45.05% (17.8–63.2) %	26.1% (−21.6 to 100) %	38.35% (0.8–100) %	0.07
Number of CDH	12	56	67	
Re-protrude	0	2	0	0.18
Unchanged	0	9	6	
Partial	1	13	12	
Median	7	18	27	
Massive	4	9	15	
Complete	0	3	9

**Table 5:** Improvement in JOA and VAS scores in CMEL-treated patients.

	Pre-operative JOA score	Post-operative JOA score	p value
1-level (n = 4)	9.3 ± 2.4	15.3 ± 1.0	0.125
2-level (n = 13)	11.9 ± 3.0	14.7 ± 1.4	< 0.001***
3-level (n = 17)	11.8 ± 2.0	14.9 ± 2.2	< 0.001***
All patients (n = 34)	11.5 ± 2.5	14.9 ± 1.8	< 0.001***

	Pre-operative VAS score	Post-operative VAS score	p value
1-level (n = 4)	1.0 ± 2.0	0.0 ± 0.0	0.99
2-level (n = 13)	1.6 ± 1.8	1.1 ± 1.3	0.031*
3-level (n = 17)	3.9 ± 2.6	1.4 ± 1.6	< 0.001***
All patients (n = 34)	2.7 ± 2.5	1.1 ± 1.4	< 0.001***

*p-value <0.05; ***p-value <0.001.

**Figure 3:**
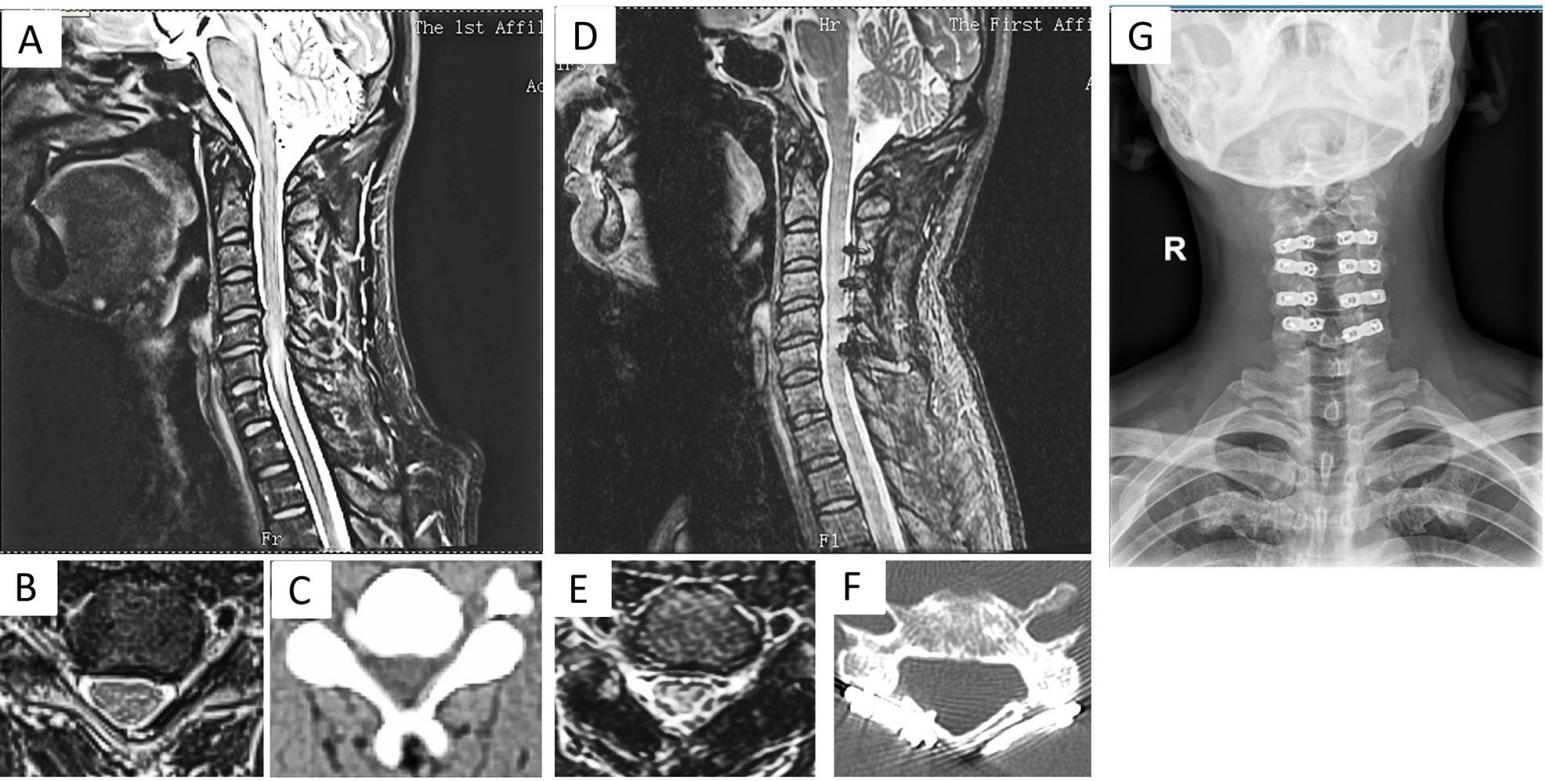
Preoperative and postoperative MRI results showing a spontaneous regression of CDH in one patient with two-level myelopathy. The represented MRI image on sagittal view, coronal view, and CT scan results pre-operatively (**A–C**) and two years post-operatively (**D–F**) demonstrated the resolution of CDH after CMEL (regress ratio of 34.9% and 34.4% for C4/5 and C5/6). (**G**) Postoperative X-ray images showed the cervical spine morphology after CMEL.

Figure 3: A patient with two-level myelopathy and stenosis. A 45-year-old male complained of limb numbness for 2 months. MRI confirmed CDH in C4/5 and C5/6 (Fig. 3A,B for C4/5). After CMEL treatment, the symptoms were alleviated. The last follow-up physical examination was conducted 24 months after surgery. The herniated discs in C4/5 and C5/6 were obviously resorbed with percentage change ratios of 34.9% and 34.4% (Fig. 3D,E for C4/5), respectively. The JOA scores were improved from 13 to 15, indicating a satisfying recovery. Bone healing and connection were seen in the CT scan results (Fig. 3F). The X-ray results confirmed bilateral plating (Fig. 3G).

**Figure 4:**
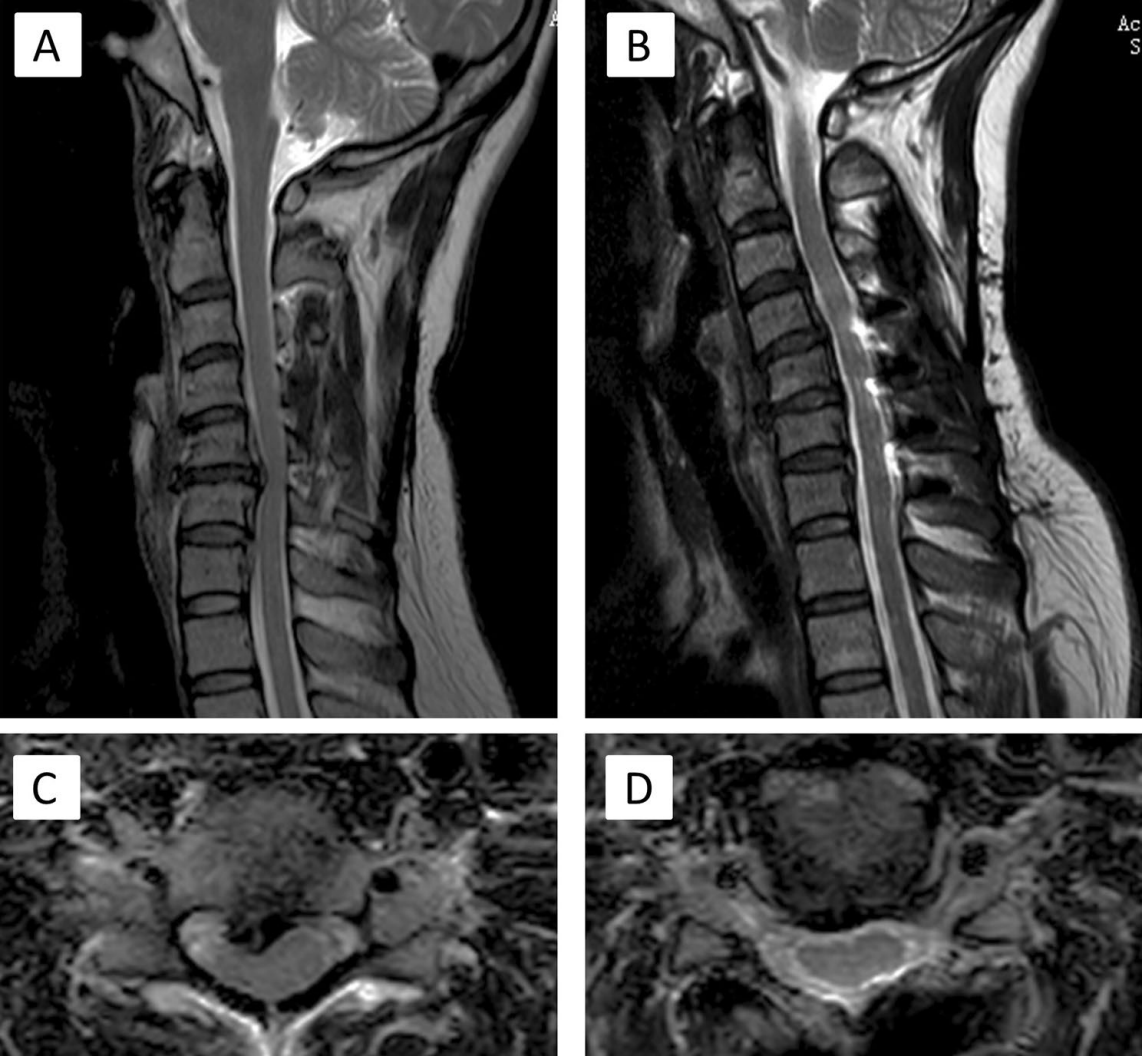
One patient with single-level myelopathy and loss of cervical lordosis showing a complete resolution of CDH. The sagittal preoperative MRI showed the C5/6 disc herniation (**A,C**) with single-level myelopathy. Then this patient accepted CMEL treatment. After 15 months, the MRI image indicated the profound regression of CDH (**B,D**).

Figure 4: A patient with single-level myelopathy and loss of cervical lordosis showing an obvious resolution of CDH after CMEL. A 43-year-old female complained of severe pain and numbness for 10 days. She visited our institution with MRI detection showing CDH located at level C5/6 (Fig. 4A,C) and loss of cervical lordotic curvature. Then, CMEL treatment was conducted. This patient revisited our institution 15 months postoperatively. She showed a complete diminishment of CDH volume and restoration of cervical lordosis to some extent as detected by MRI (Fig. 4B,D).

## Discussion

In this prospective study, we first showed that in DCM patients with 1- to 3-level myelopathy, CMEL induced diminishment of CDH that was clinically correlated with the restoration of neurological function (JOA score). This phenomenon indicated the possibility that indirect removal of CDH, either partial or complete, was achieved by surgical CMEL. Considering this point, the combined or subsequent anterior dissection of disc herniation by operations, e.g., corpectomy and fusion, could be avoided to some extent in patients with 1- to 3-level myelopathy. This study therefore suggested a novel therapeutic strategy for DCM.

The CDH we focused on in the present study was the disc tissue that migrated into the spinal canal/compressing the spinal cord or nerve root on sagittal MRI views. It mainly contained nucleus pulposus without any radiographic sign of calcification or ossification. These characteristics gave the CDH type we cared about both relationship to clinical symptoms and capacity to resolve. This study utilized the three-dimensional (3D) measurement of MRI-derived CDH volume instead of other indices previously reported, such as the area of CDH in relationship to the spinal canal^13^, which only provided partial information. Many studies^12,14^ have used a very similar method to calculate the disc herniation volume, but they did not show how large the manual error could be. We thus identified the range of manual measurement error within ± 5% (maximum of 4.52%) using this 3D calculation method to calculate the randomly placed 5 ml saline on MRI images. To carefully define the unchanged condition of CDH volume, a twofold range of manual error was obtained (−10% to 10%).

Surgical management of DCM mainly includes anterior and posterior techniques, and efforts have been made in numerous studies^15,16^, to identify the difference between the two approaches. In some studies, patients displayed no significant inter-approach differences regarding quality of life and rates of complications and reoperation. In contrast, in one cohort study, laminoplasty was associated with better clinical scores than corpectomy^17^. In view of this study, we suggested that DCM patients with multilevel or even single- to three-level myelopathy/stenosis were proper candidates to receive the laminoplasty of CMEL in a posterior approach. The advantage of laminoplasty over corpectomy is avoiding complications associated with operation and arthrodesis, such as wound problems, laryngeal oedema, restricted range of motion, and adjacent disc degeneration^18^. The laminoplasty-associated preservation of cervical alignment and the range of motion also contribute to recovery^19^. According to current knowledge, laminoplasty is a nonfusion procedure to enlarge the spinal canal, which indirectly decompresses the anterior component of compression on the spinal cord. Our results deepen the understanding that bilateral and asymmetric laminoplasty (CMEL) induce the spontaneous resolution of CDH.

DCM patients with single- or two-level myelopathy were best candidates for anterior corpectomy and fusion, especially with the existence of kyphosis. The ideal indication for laminoplasty was multilevel stenosis (generally 3 or more segments), with lordosis and minimal spondylotic axial pain. Indeed, the myelopathy levels were not rigid criteria for operation selection. Anterior cervical discectomy and fusion were also applied in multilevel segments (3 or 4 continuous levels)^20^; a few cases were reported of laminoplasty as treatment for single/two-segment myelopathy^21^. In this study, the number of affected CDH levels did not influence the clinical outcomes or the reduction in CDH volume in CMEL-treated patients. The VAS score from the involved patients varied, ranging from 0 (35.3%) to 8 (5.9%). Most of the patients scored moderate to zero in pain, but serious pain was also alleviated by CMEL. Notably, several patients with kyphosis who accepted CMEL in the posterior approach gradually recovered with the preservation of sagittal alignment, also suggesting that kyphosis is not a major contraindication of CMEL. This was consistent with a previous study showing that cervical curvature was not the determiner of spinal cord shift by laminoplasty^11^. A conventional interpretation illustrated that the shift of the spinal cord involved release from alignment with cervical lordotic curvature and then straightening. According to this theory, laminoplasty requires multiple levels to guarantee a spinal cord shift. Our results were contrary to this point because a satisfactory spinal shift was obtained in 2- or 3-level CMEL cases. The correlation study showing shift of the dural sac as the major factor associated with decompression^10^ accounted for our observation. These observations widen the understanding of the clinical application of laminoplasty/CMEL.

The improvement in the JOA score in this prospective study was found to be acceptable, with a moderate change from 11.5 ± 2.5 to 14.9 ± 1.8. This was consistent with previous studies. For example, in one systematic review of 28 cohort studies, the JOA score was 14.1 (11.4–15.7), which improved from 10.1 (5.8–14.2) preoperatively^22^. We also found a significant relationship between improvements in JOA scores and CDH regression. This was probably due to the remaining pressure of CDH against the spinal cord/nerve root or the progression of cervical degeneration after surgery. CDH regression further attenuated the possibility of compression and neurological symptoms, thus conferring a positive correlation. The VAS scores surveyed in this study were low, approximately 1–3 on average. This was because DCM patients visited our institution mostly due to neurological symptoms such as numbness and muscle weakness, not pain. In terms of the range of motion (ROM), we previously performed X-ray detection, but no significant difference was found^6^. The preoperative ROM (41.08° ± 13.68°) did not significantly differ from the postoperative ROM (44.68° ± 11.38°).

The spontaneous resolution of CDH has been presented previously in some cases and has been shown to be correlated with clinical symptom alleviation^14^. This kind of regression is common in the lumbar region, which has been documented and explained in terms of the macrophage-mediated inflammatory response in the outermost layer of the herniation^23–25^. The term resorption of herniated nucleus pulposus (RHNP) was proposed to reflect this kind of resolution. Unlike this gradual inflammatory response, which is slow, the regression of CDH in our study was pronounced 7 days postoperatively. Considering the decompression of CDH after CEML, we herein proposed that the regression of CDH is likely due to the withdrawal of mechanical compression regulating extracellular matrix turnover^26^ or other biochemical factors. Because this kind of CDH regression was achieved by manual surgical treatment and occurred in a relatively rapid way, we preferred to explain it as the phenomenon of induced RHNP (i-RHNP). The underlying mechanism of i-RHNP further needs to be illustrated.

The limitations of the present study include the lack of comparison to other surgical treatments, such as classic open-door laminoplasty and anterior cervical discectomy and fusion. Because of the proposed mechanism relative to compression withdrawal, it is likely that other examples of CDH regression may be achieved by various treatments that decompress CDH. Further studies are needed to show the role of CDH regression in clinical recovery and elucidate the molecular mechanism of CDH volume regression.

## Data Availability

All the raw MRI images, clinical outcomes and analysis results used during the current study available from the corresponding author on reasonable request. We did not share the raw data along this paper as a patent was prepared.
